# Cereal leaf beetle‐associated bacteria enhance the survival of their host upon insecticide treatments and respond differently to insecticides with different modes of action

**DOI:** 10.1111/1758-2229.13247

**Published:** 2024-04-21

**Authors:** Beata Wielkopolan, Alicja Szabelska‐Beręsewicz, Jan Gawor, Aleksandra Obrępalska‐Stęplowska

**Affiliations:** ^1^ Department of Monitoring and Signaling of Agrophages Institute of Plant Protection–National Research Institute Poznan Poland; ^2^ Department of Mathematical and Statistical Methods Poznań University of Life Sciences Poznan Poland; ^3^ DNA Sequencing and Synthesis Facility Institute of Biochemistry and Biophysics, Polish Academy of Sciences Warsaw Poland; ^4^ Department of Molecular Biology and Biotechnology Institute of Plant Protection–National Research Institute Poznan Poland

## Abstract

The cereal leaf beetle (CLB, *Oulema melanopus*) is one of the major cereal pests. The effect of insecticides belonging to different chemical classes, with different mechanisms of action and the active substances' concentrations on the CLB bacterial microbiome, was investigated. Targeted metagenomic analysis of the V3–V4 regions of the 16S ribosomal gene was used to determine the composition of the CLB bacterial microbiome. Each of the insecticides caused a decrease in the abundance of bacteria of the genus *Pantoea*, and an increase in the abundance of bacteria of the genus *Stenotrophomonas*, *Acinetobacter*, compared to untreated insects. After cypermethrin application, a decrease in the relative abundance of bacteria of the genus *Pseudomonas* was noted. The dominant bacterial genera in cypermethrin‐treated larvae were *Lactococcus, Pantoea*, while in insects exposed to chlorpyrifos or flonicamid it was *Pseudomonas*. Insecticide‐treated larvae were characterized, on average, by higher biodiversity and richness of bacterial genera, compared to untreated insects. The depletion of CLB‐associated bacteria resulted in a decrease in larval survival, especially after cypermethrin and chlorpyrifos treatments. The use of a metagenome‐based functional prediction approach revealed a higher predicted function of bacterial acetyl‐CoA C‐acetyltransferase in flonicamid and chlorpyrifos‐treated larvae and tRNA dimethyltransferase in cypermethrin‐treated insects than in untreated insects.

## Graphical abstract

Depletion of cereal leaf beetle (CLB)‐associated bacteria resulted in reduced survival of CLB larvae treated with insecticides. The biodiversity of bacterial genera associated with CLB changed depending on the class (and its mode of action) and the insecticide dose. *Acinetobacter*, *Stenotrophomonas*, *Lactococcus* and *Pseudomonas* are the bacterial genera potentially involved in reducing the harmful effects of the tested insecticides on CLB larvae.

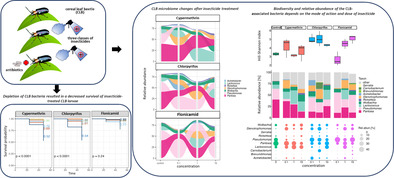

## INTRODUCTION

Cereal leaf beetle (CLB, *Oulema* spp., Coleoptera, Chrysomelidae) is one of the major herbivorous insect pests causing significant yield losses, among others in wheat, barley or oat cultivation (Buntin et al., 2004; Ihrig et al., 2001; Steinger et al., 2020; Walczak, 2010). It was estimated that a single larva of CLB is able to reduce plant assimilation by about 10% (Heyer, 1977), whereas massive feeding of this pest can reduce total assimilation by up to 80% (Campbell et al., 1989; Tanasković et al., 2012). Chemical treatment still plays a primary role in the control of CLB. Many active substances of insecticides (including cypermethrin, deltamethrin, chlorpyrifos, flonicamid or acetamiprid) were registered against CLB larvae, in Poland.

Currently, more than 3.5 million tons of pesticides are used throughout the world, of which 29.5% are insecticides (Jaffar et al., 2022; Sharma et al., 2019) that are widely used to reduce crop losses. Their excessive use contaminates the flora, and ecosystem (plants, air, water, soil) and has a negative impact on non‐target living organisms, including beneficial fauna (pollinators and predators of pests) (Jaffar et al., 2022; Mallinger et al., 2015; Obregon et al., 2021; Zhao et al., 2022). Due to the indiscriminate use of insecticides over the years, herbivorous insects, including CLB, have developed insecticide resistance (Tanasković et al., 2012). Several beetles adaptations have been identified to explain the reduced efficiency of insecticides, including (a) mutation of insecticide action sites (Obrępalska‐Stęplowska et al., 2016; Wrzesińska et al., 2014), (b) thickening of insect epidermis reducing insecticide penetration (Hemingway & Karunaratne, 1998; Rocha et al., 2021; South & Hastings, 2018; Wang et al., 2022), (c) increase in insecticide metabolism through up‐regulation of genes encoding enzymes such as cytochrome P450, monooxygenase, glutathione S‐transferase, carboxylesterase, acetylcholinesterase, hydrolase and many more associated with insecticide degradation (Chang et al., 2017; Cisse et al., 2015; Jaffar et al., 2022; Vontas et al., 2020; Wang et al., 2022; Wieczorek et al., 2014). The implication of symbiotic microbes as potential contributors to insecticide resistance is of great interest. This is evidenced by the growing number of publications addressing this topic. It was indicated that enzymes encoded by genes of the insect's gut microbiome can participate in the adaptability of insects to insecticides (Blanton & Peterson, 2020). Insect‐associated microbes are able to utilize insecticides as a sole source of carbon and energy (Jaffar et al., 2022), but the ability to degrade insecticides depends, among others, on the temperature, pH, nutrients availability, structure of a bacterial community or class of the insecticide (Gomes et al., 2020). It was recorded that bacteria might detoxify multiple insecticide classes including pyrethroids, neonicotinoids, organophosphates (Blanton & Peterson, 2020; Stara & Hubert, 2023). For instance, *Burkholderia mallei*, *Stenotrophomonas maltophilia* or *Citrobacter amalonaticus* can enhance the host's metabolism of insecticides (Wang et al., 2022). Generally, it was noted that the following genera of bacteria: *Pseudomonas*, *Bacillus*, *Rhodococcus*, *Stenotrophomonas*, *Alcaligenes*, *Serratia* and *Streptomyces* might participate in the degradation of various pesticides (Özdal & Algur, 2022).

The diversity of bacterial communities depends on many factors, including insect host species (Bonilla‐Rosso & Engel, 2018; Wielkopolan et al., 2021), developmental stage (Cini et al., 2020; Wielkopolan et al., 2021), diet, and environmental conditions (Kolasa et al., 2019). Also, insecticides used in pest control can significantly change the structure and function of the bacteria inhabiting the insect's gut (Giglio et al., 2021; Syromyatnikov et al., 2020) thus affecting insect response to environmental stress factors.

Previous targeted metagenomic analysis has highlighted that the CLB developmental stage, the host plant and the insect's sampling location affected the CLB's bacterial microbiome structure, biodiversity and richness (Wielkopolan et al., 2021). However, studies addressing the contribution of the bacteria to CLB insecticide resistance are currently not available. Therefore, the main aim of the study was to determine the possible involvement of the CLB bacterial microbiome in the reduced efficacy of insecticides used against CLB larvae. We tested the following hypotheses (i) bacterial microbiome increases the chances of survival of CLB larvae after insecticide treatment, (ii) different genera of bacteria may be involved in the detoxification process of different insecticides with different mechanisms of action. To test our hypotheses, we evaluated the impact of three classes of insecticides (containing different active substances: chlorpyrifos, cypermethrin, flonicamid) with different modes of action, on structure, richness, diversity and function of bacteria of CLB larvae and on larval survival. We have chosen these insecticides due to their long period of use in crop protection. Cypermethrin is a synthetic pyrethroid used as an insecticide in large‐scale commercial agricultural applications, and is a fast‐acting neurotoxin in insects, disturbing the functioning of the voltage‐gated sodium channels (Özdal & Algur, 2022). Chlorpyrifos is an organophosphate pesticide, that is an inhibitor of acetylcholinesterase, that causes acute neurotoxicity. Chlorpyrifos is one of the most widely used pesticides worldwide, except in the European Union, where its use was banned in 2020 (Stara & Hubert, 2023). In turn, flonicamid, is a pyridinecarboxamide (Zhao, Wang, et al., 2021), that disrupts insect chordotonal organs, but the specific target site of the chemical is unknown (Spalthoff et al., 2022).

The Illumina MiSeq sequencing of the V3–V4 hypervariable regions of the bacterial 16S ribosomal gene (16S rRNA) was used to identify the genera of bacteria and their abundance depending on the treatment studied. The structure of the bacterial community in larvae treated with insecticides differed markedly from those not treated by insecticide (control). Insecticide treatment of larvae led to changes in dominant bacterial genera and to the restructuring of the bacterial community. We found out that the abundance of some of the bacterial genera including *Acinetobacter*, *Stenotrophomonas*, *Pseudomonas*, and *Lactococcus* was significantly higher in insecticide‐treated larvae (depending on the class of insecticide), compared to the control group of insects. Also, a higher level of biodiversity of bacterial genera was recorded in insecticide‐treated larvae than in untreated insects.

This study makes a significant contribution to understanding the role of CLB larvae‐associated bacteria in insecticide detoxification.

## EXPERIMENTAL PROCEDURES

### Insect collection and insecticide treatment

CLB larvae (size 3 mm), were collected from a winter wheat field in Słupia Wielka (52°13′02″ N 17°13′04″ E), in 2021. Larvae were starved for 24 h before insecticide treatments, and then were placed on previously disinfected wheat leaves (rinsed once in 70% ethanol, three times in sterile distilled water to wash the ethanol residues) on Petri dishes with 1% agarose medium (so that the leaves retained their moisture). Next, larvae were sprayed with the following three classes of insecticides (separately): pyridinecarboxamide (flonicamid), organophosphate (chlorpyrifos), pyrethroid (cypermethrin). Three concentrations (0.1 μg/mL; 1 μg/mL; 10 μg/m) of active substances of insecticides were used. Insecticide treatment was performed in the cabinet, the following parameters were used: speed 3.16 km/h, pressure on the cylinder 1.5 bar, a dose of liquid 200 L/ha, output l/min‐0.79 L/min. CLB larvae not treated with insecticide were considered as the control. Twenty larvae were used per treatment. Two days after the insecticide treatment larvae were placed in 70% ethanol and stored at −20°C until DNA isolation.

To understand the influence of bacteria on the survival rate of larvae upon insecticide treatment, the insects were treated with a mixture of antibiotics (AB) and compared to the untreated ones. The AB mixture (0.003 g streptomycin sulfate, 0.05 g chlortetracycline in 50 mL of autoclaved distilled water) was placed on a previously disinfected wheat leaf (washed once in 70% ethanol, three times in sterile distilled water) on Petri dishes with 1% agarose medium. After 24 h, the larvae were exposed to the above‐mentioned classes of insecticides at three concentrations of the active substance (0.1 μg/mL; 1 μg/mL; 10 μg/mL). Insecticide treatments were performed according to the parameters mentioned above. The controls were AB‐treated insects and untreated insects (neither AB nor insecticide). Twenty larvae were used per treatment, in duplicate. The survival rate of CLB larvae was assessed at 24 and 48 h after treatment with an insecticide.

### DNA extraction

Prior to DNA extraction, each CLB larva was rinsed three times with sterile distilled water in 2.0 mL Eppendorf tubes to remove contaminations. DNA was individually isolated from the whole CLB larvae by using a Genomic Mini AXE kit (A&A Biotechnology) with Proteinase K, lysozyme, mutanolysin and with RNase treatment step. DNA was resuspended in 40 μL of 10 mM Tris–HCl buffer, pH 8.5. The quantity and quality of the DNA were measured with a NanoDrop 2000C spectrophotometer (Thermo Scientific, USA). Each treatment consisted of three biological samples (each sample consisted of DNA pooled from two larvae). Blank (isolation according to the protocol without the addition of the insect) was performed as a control for monitoring contamination of environmental bacteria DNA.

### Amplification and sequencing of the V3‐V4 region of the 16S rRNA

Bacterial identification of CLB larvae was done by 16S rRNA gene sequencing at the V3–V4 hypervariable region by using next generation sequencing (NGS). Amplicon libraries and sequencing was done by CeGat GmbH (Germany), basically as described in (Wielkopolan et al., 2021). Amplicon libraries for each of the samples were prepared using the Quick‐16S NGS Prep Kit (Zymo Research). The library was sequenced on an Illumina MiSeq instrument using Nano V2 chemistry kit. Twenty nanograms of DNA per sample were used for sequencing. The positive and negative controls of the sequencing process were also done. Data from 31 samples were obtained independently.

### Bioinformatic analysis, taxonomic classification

Sequence reads were quality‐checked using the FastQC toolkit (Andrews, 2010) and processed using the Qiime2 pipeline (Bolyen et al., 2019). Paired reads were merged and denoised using DADA2 (Callahan et al., 2016). Sequences were then taxonomically classified using a Bayesian Naïve classifier pre‐trained on the SILVA database ‘silva‐138‐99‐nb‐classifier.qza a’ (Quast et al., 2013). The SILVA database was also used to remove chloroplast and mitochondrial DNA containing reads. Downstream analysis was performed in the R environment (R Core Team, 2022). Statistical analysis and visualization were performed using the following packages: phyloseq (McMurdie & Holmes, 2013), microbiome (Lahti & Shetty, 2023), ggplot2 (Wickham, 2016) and Ampvis2 (Andersen, 2023; Andersen et al., 2018). For statistical analyses, the singletons, defined as the taxa observed in less than 2 samples, were excluded.

### Survival rate analysis

Survival rate analysis was performed using R package survival (Therneau & Grambsch, 2000). For each group determined based on the variables concerning ‘the active substance’, its ‘concentration’ as well as usage of antibiotics the Kaplan–Meier curves (Turnbull, 1974) were created and compared using R package survminer (Kassambara et al., 2021).

### Relative abundance

The genera with a relative abundance >1% were presented using boxplots, bar plots and heatmap together with hierarchical clustering with method Ward (Murtagh & Legendre, 2014) including information about the active substance of insecticide and their concentration. In addition, nonmetric multidimensional scaling (NMDS) analysis (Minchin, 1987) was performed using R package vegan (Oksanen et al., 2022) to compare and evaluate differences between bacterial communities depending on the variable ‘the active substance of insecticide’ and its ‘concentration’.

### Differential abundance analysis

Differential abundance analysis with the usage of DESeq2 (Love et al., 2014) method implemented in R package animalcules (Zhao, Federico, et al., 2021) was performed to indicate bacteria genera that were characterized by a significantly different number of counts in insecticide‐treated CLB larvae for the three concentrations of the active substance of insecticide used, compared to the control group of insects. The results are presented in the form of a heatmap and a table.

### Alpha biodiversity coefficient

Bacterial biodiversity was assessed using several identifiers. Alpha biodiversity, which describes the richness and uniformity of the bacterial community in the sample, was determined using Hill numbers (Hill, 1973). The first measure used is the Hill number of order 1, which is closely related to the Shannon index (Shannon & Weaver, 1964). It will be referred to as the Hill‐Shannon index throughout the text. To determine the level of biodiversity within each variable considered, the Hill number of order 0 was used. It is closely related to species richness (Chao, 1984). The higher the value, the higher the biodiversity. It will be referred to as the Hill richness index throughout the text. The calculations of the indices were done with R package iNEXT (Chao et al., 2014). Based on these measures, a non‐parametric Kruskal‐Wallis test (Chao et al., 2014), as well as a pairwise Wilcoxon Rank Sum test (Bauer, 1972) for variables of ‘the active substance of insecticide’ and its ‘concentration’ were used to determine differences between considered groups.

### Beta biodiversity coefficient

Permutational multivariate analysis of variance (PERMANOVA) (McArdle & Anderson, 2001) available in vegan package (Oksanen et al., 2022) was performed for beta diversity. It allows for the determination of significant differences in the level of biodiversity measured by the beta coefficient. The distance matrices needed for the procedure were calculated using Bray‐Curtis metrics (Bray & Curtis, 1957). The obtained p‐values were calculated in permutational procedure with 1000 permutations.

### Modelling the structure of CLB larvae bacterial microbiome with generalized linear mixed model

To determine the influence of multiple factors, the abundance of bacteria was modelled with a negative binomial generalized linear mixed model (NB GLMM). In the model, ‘the active substances of insecticides’ and their ‘concentrations’ were used as fixed variables. Taxonomic rank was included as a random effect. Parameter estimation and model fitting were performed using methods implemented in the R package glmmTMB (Brooks et al., 2017). The significance of fixed effects was verified with the Wald II type test (Fox, 2016). Next, the multiple comparisons procedure based on Tukey's test (Bretz et al., 2010) implemented in R package multcomp (Hothorn et al., 2008) was applied to obtain the information on which levels of active substance and concentration are significantly different from each other. The Top Biome, defined as those bacterial genera whose abundance is significantly different from the overall mean abundance for all genera, was determined by calculating the 99.9% confidence intervals for each analysed genus, which was considered a random variable in the model effect.

### Functional prediction of CLB larvae microbiota

Prediction of functional abundances based on 16S marker gene sequences was performed using Picrust2 software with default parameters (Douglas et al., 2020). The STAMP software package (version 2.1.3) (Parks et al., 2014) was employed to determine differentially enriched metabolic pathways (*p* < 0.05) and their effect sizes (<0.8) using one‐way analysis of variance (ANOVA).

Picrust2 predicted and manually selected data for Kyoto Encyclopedia of Genes and Genomes (KEGG) ortholog (KO) families, Clusters of Orthologs Groups (COGs) and metabolic pathways were further analysed and visualized using MicrobiomeAnalyst 2.0 (Lu et al., 2023) and R package pheatmap (Kolde, 2019).

## RESULTS

### Illumina sequencing data and taxonomic assignments

Illumina sequencing yielded a total of 2,156,350 raw reads, with an average of 94,780 ± 37,302 raw reads per sample. After quality trimming, filtering and downstream analyses 963,412 high‐quality sequences were obtained, with an average of 32,114 sequences per sample. The number of sequences varied for the samples (max. 42,105, min. 16,917). In the case of the blank sample, 184 raw reads were obtained, from which, after data processing 67 high‐quality sequences were obtained (Table S1).

Next, high‐quality sequences were classified into the bacterial taxa using the SILVA database implemented in Qiime2 software. Obtained OTUs were summed within the rank, and assigned to 6 bacterial phyla, among them, 8 classes, 25 orders, 42 families and 54 genera were determined for all tested insect samples. After removing the singletons, the 5 phyla, 6 classes, 17 orders, 29 families and 32 genera were determined.

Rarefaction curves calculated for a total number of reads aggregated at the genus level and assessed using the maximum likelihood phylogeny analysis always reach the plateau indicating adequate sequencing depth to analyse (Figure S1).

### Depletion of CLB bacteria by antibiotics resulted in a decreased survival of insecticide‐treated CLB larvae

**FIGURE 1:**
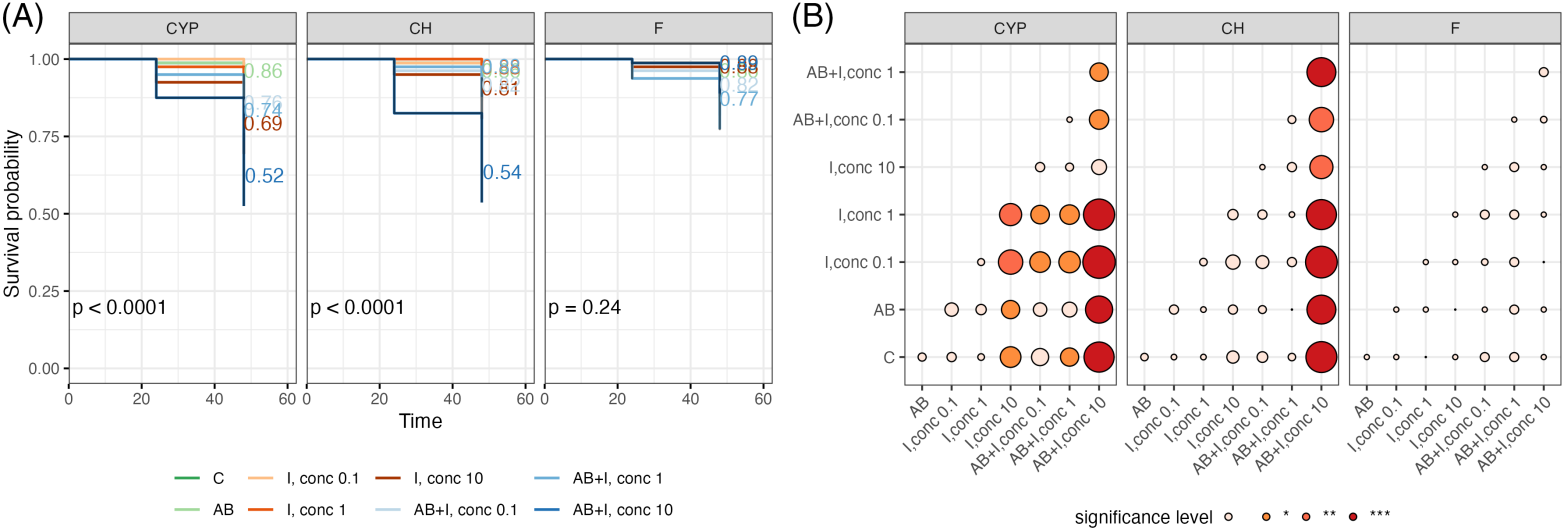
(A) Kaplan–Meier curve depicting the survival probability of cereal leaf beetle (CLB) larvae in response to antibiotics (AB) and to insecticide (I) treatment at concentration of the active substance of insecticide 0.1, 1 and 10 μg/mL (cypermethrin [CYP], chlorpyrifos [CH], flonicamid [F]), during the course of 48 h experiment (20 larvae per treatment, two replicates for each treatment). The controls (C) were AB‐treated larvae and untreated insects (neither AB nor insecticide) (B) Statistically significant differences in larval survival between pairs of treatments. Significant codes for *p*‐value: *p < 0.1, **p < 0.01, ***p < 0.001.

CLB larvae with natural intestinal microbiota (AB‐untreated) and with a reduced number of bacteria (AB‐treated) were exposed to three classes of insecticides: pyridinecarboxamide (flonicamid), organophosphate (chlorpyrifos), pyrethroid (cypermethrin) at 0.1, 1 and 10 μg/mL concentration of the active substance. The larval survival was recorded during the 48 h experiment (Figure 1A). The laboratory assay proved significant differences in the susceptibility of CLB larvae to tested insecticides. As expected, a lower survival rate was recorded in insecticide‐treated insects compared to the control group. Bacteria depletion significantly reduced the survival of larvae treated with insecticides, especially after chlorpyrifos and cypermethrin application, compared to insects treated with insecticide alone (insects with natural intestinal microbiota). The lowest percentage of larval survival was noted in cypermethrin‐treated larvae, while the highest was in the flonicamid‐treated insects (whether they were treated with AB or not) (Figure 1A).

In the case of cypermethrin and chlorpyrifos, the largest statistically significant differences (*p*‐values <0.001) in the survival rate were observed between AB, insecticide‐treated larvae (especially after using the highest concentration of the active substance) and insects treated with the insecticide alone. In turn, after flonicamid application, no statistically significant differences between survival curves with AB‐treated and AB‐untreated larvae were noted (Figure 1B).

### Insecticides affect the structure of the bacterial microbiome of the CLB larvae

#### Biodiversity and relative abundance of bacterial genera depending on the active substance of insecticide and its concentration

The composition of the microbiome was modelled with NB GLMM. The influence of the analysed variables was determined with the Wald type II test. According to the test result the variable ‘the active substance of insecticide’ and its ‘concentration’ had a statistically significant impact (*p* < 0.001) on the bacterial abundance in the CLB larvae microbiome at the genus level.

Next, differences in bacterial abundances between combinations of the applied active substance of insecticide and concentration were analysed using Tukey's test in multiple comparison. The most significant differences (*p* < 0.001) in average abundance were observed when flonicamid‐treated insects with a concentration of 10 μg/mL were compared with other groups of insects. The differences in the mean value of a genus of bacteria abundance are presented in Table S2.

**FIGURE 2:**
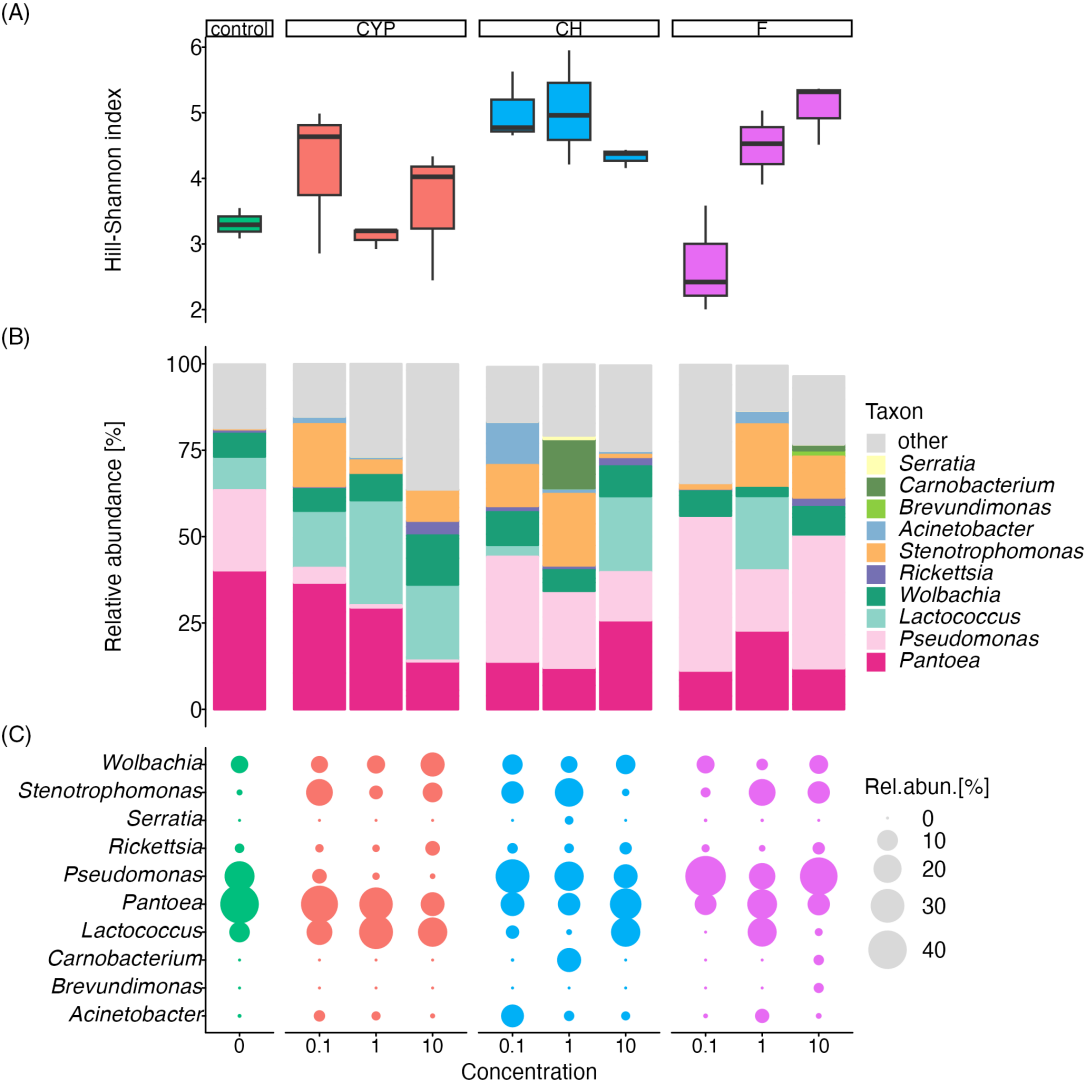
(A) Bar plots of the Hill–Shannon index observed in tested groups of cereal leaf beetle (CLB) larvae; (B) the composition and average relative abundance of bacterial genera in each group of CLB larvae depending on the active substance of insecticide (CYP, cypermethrin; CH, chlorpyrifos; F, flonicamid) and its concentration (0.1; 1; 10 μg/mL). (C) Indicator genera identified by both linear discriminant analysis of effect size and multi‐level pattern analysis. Only the taxa with a relative abundance >1% were listed, taxon comprising less than 1% were merged into other. Control – no insecticide‐treated larvae.

To investigate changes in microbial community diversity between control and insecticide‐treated groups of insects, the relative abundance profiles obtained by 16S rRNA amplicon sequence were determined. The alpha diversity of the bacterial community within the treated and control group across different concentrations of the active substances of insecticides was characterized by the Hill‐Shannon index. On average, higher bacterial biodiversity was recorded in insecticide‐treated larvae (with two exceptions: cypermethrin at a concentration of 1 μg/mL and flonicamid at 0.1 μg/mL) compared to the control. In the case of flonicamid, an increase in the biodiversity of bacterial genera was noted with an increase in the concentration of this active substance of insecticide. A markedly higher biodiversity of bacterial genera was observed in chlorpyrifos‐treated larvae, for each of the applied concentrations of the active substance (Figure 2A).

**FIGURE 3:**
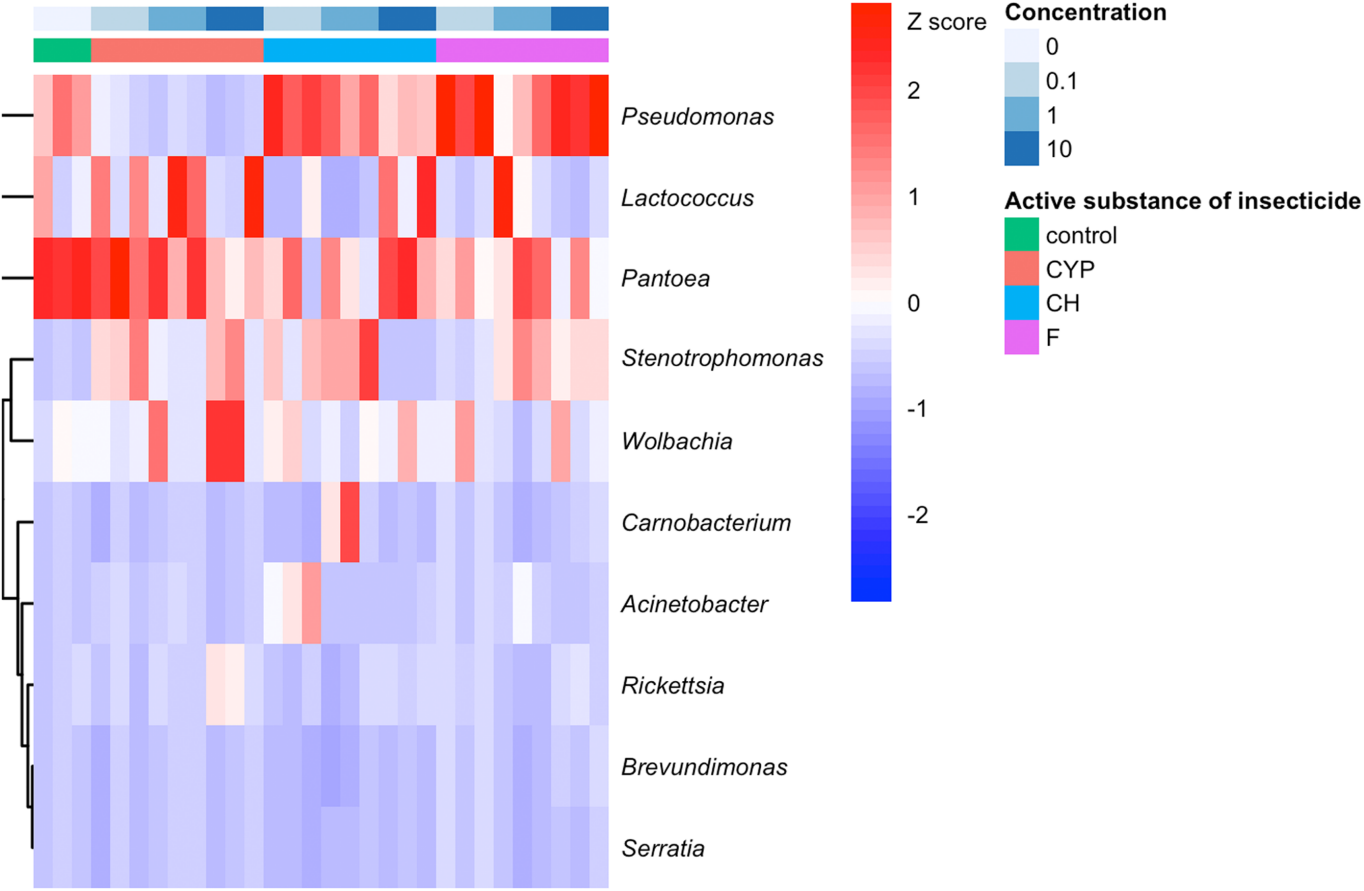
Heatmap analysis of the standardized relative abundance of bacterial genera associated with cereal leaf beetle (CLB) larvae depending on the variable ‘the active substance of insecticide’ (CYP, cypermethrin; CH, chlorpyrifos; F, flonicamid) and its ‘concentration’ (0.1; 1; 10 μg/mL). Only the taxa with relative abundance >1% were listed. Control – no insecticide‐treated larvae.

The structure of the bacterial community in insecticide‐treated larvae differed markedly from those untreated (neither AB nor insecticide). Insecticide treatment of larvae led to changes in dominant bacterial genera and to the restructuring of the bacterial community. The most abundant genera of bacteria in the untreated group of larvae were *Pantoea* (40.25%) and *Pseudomonas* (23.17%). After the application of cypermethrin (for all concentrations used) the relative abundance of *Lactococcus* sharply increased, while *Pseudomonas* decreased, compared to untreated larvae. Contrary to larvae exposed to chlorpyrifos and flonicamid where an increase in the relative abundance of the *Pseudomonas* genus was noted (depending on the concentration of the active substance), compared to the control. The relative abundance of *Lactococcus* sharply increased after chlorpyrifos and flonicamid application at the concentrations 10 and 1 μg/mL, respectively. The dominant bacterial genera in cypermethrin‐treated larvae were *Lactococcus* and *Pantoea*, while in flonicamid‐ and chlorpyrifos‐exposed larvae it was *Pseudomonas*. After the application of each of the active substances of insecticides for each of the three tested concentrations an increase in the abundance of bacteria of the genus *Stenotrophomonas* and *Acinetobacter* was noted, compared to the control. After treatment with chlorpyrifos at 10 μg/mL concentration an increase in the relative abundance of bacteria belonging to the genera *Carnobacterium* and *Erwinia* was observed (Figures 2B, C and 3; Figure S2).

#### Genera of bacteria affected by three classes of insecticides

**FIGURE 4:**
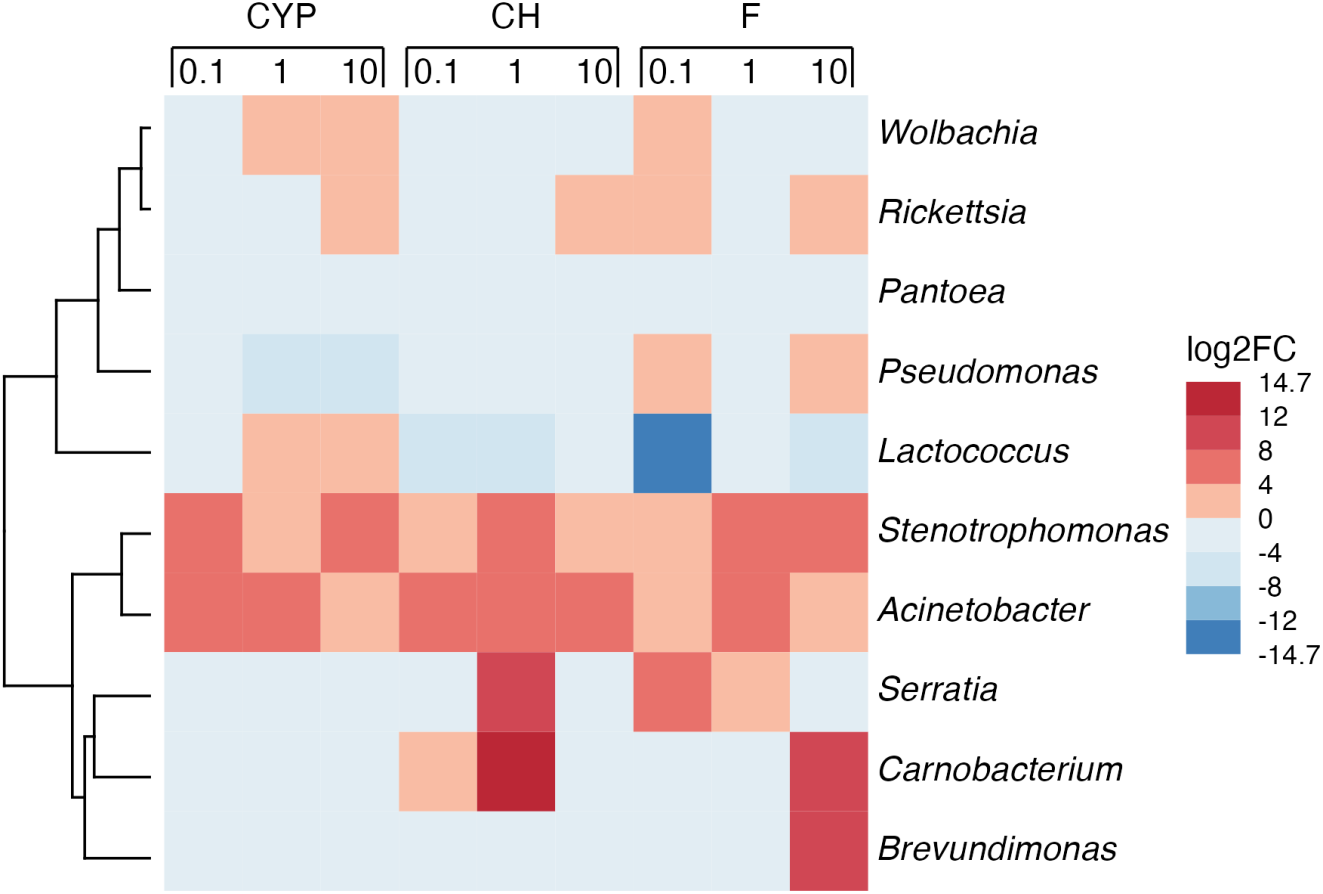
The differential abundance analysis DESeq2 of counts of cereal leaf beetle (CLB) larvae‐associated bacterial genera affected by three classes of insecticides containing the following active substances: cypermethrin (CYP), chlorpyrifos (CH) and flonicamid (F) at 0.1, 1 and 10 μg/mL concentration in comparison to the no insecticide‐treated larvae (control). Only the taxa with relative abundance >1% were listed.

The differential abundance analysis DESeq2 was performed to identify bacterial genera affected by three classes of insecticides at 0.1, 1 and 10 μg/mL concentration of the active substances in comparison to the untreated group of insects (control). Statistical analyses showed that the number of counts for the genus *Stenotrophomonas* for each of the insecticides used was significantly (*p* < 0.001) higher than in the control group of insects (except for chlorpyrifos at a concentration of 10 μg/mL, where no statistically significant differences were noted). Exposure to the applied insecticides had also a significant (*p* < 0.001) effect on the number of counts of the bacteria belonging to the *Acinetobacter* (higher number of counts for chlorpyrifos at 0.1 and 1 μg/mL; cypermethrin at 0.1 and 1 μg/mL; flonicamid at 1 μg/mL), *Ralstonia* (lower number of counts for chlorpyrifos at 0.1 μg/mL) and *Lactococcus* (lower number of counts for flonicamid at 0.1 μg/mL) genera. The results are presented in the form of a heatmap (Figure 4 and Table S3).

#### Modelling the structure of the bacterial microbiome of CLB larvae with GLMM

**FIGURE 5:**
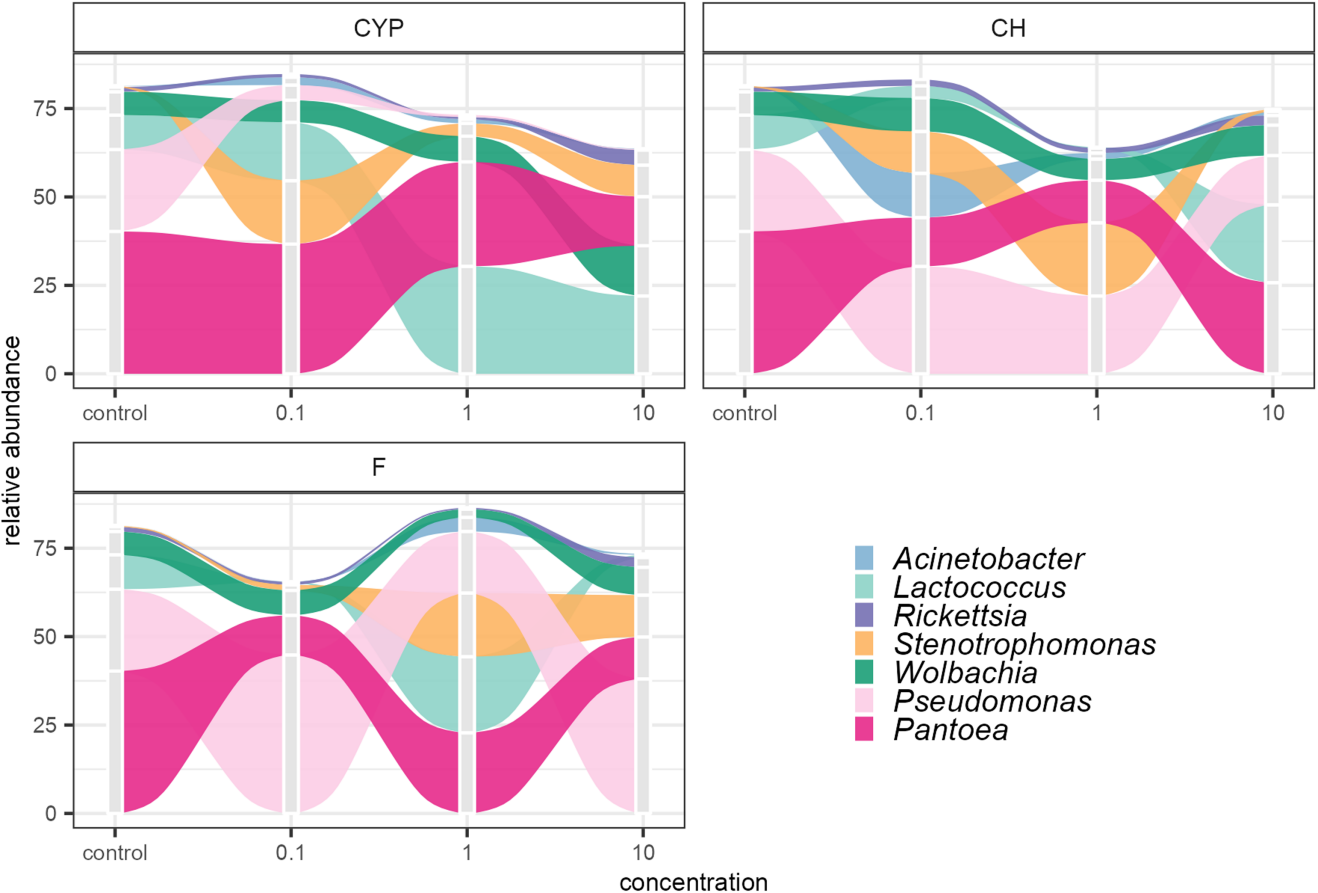
Distribution of cereal leaf beetle (CLB) larvae – associated bacteria genera depending on the variable ‘the active substance of insecticide’ (CYP, cypermethrin; CH, chlorpyrifos; F, flonicamid) and its ‘concentration’ (0.1; 1; 10 μg/mL) with respect to the no insecticide‐treated larvae (control). The rank taxa with the highest mean value of relative abundance for taxa from the Top Biome on each taxonomic level are included in the graph.

The set of bacterial genera denoted as Top Biome was determined based on NB GLMM for all tested treatments together (insecticide and no insecticide‐treated larvae with considered concentration levels). The abundance of the following seven bacteria genera: *Pantoea*, *Pseudomonas*, *Wolbachia, Stenotrophomonas*, *Rickettsia*, *Lactococcus*, *Acinetobacter* was significantly different from the overall mean abundance for all genera with respect to control (Figure 5).

### The active substances of insecticides and their concentrations affect the biodiversity of bacterial genera of CLB larvae

#### Analysis of the biodiversity of CLB larvae‐associated bacteria in reference to the insecticide active substance used

**FIGURE 6:**
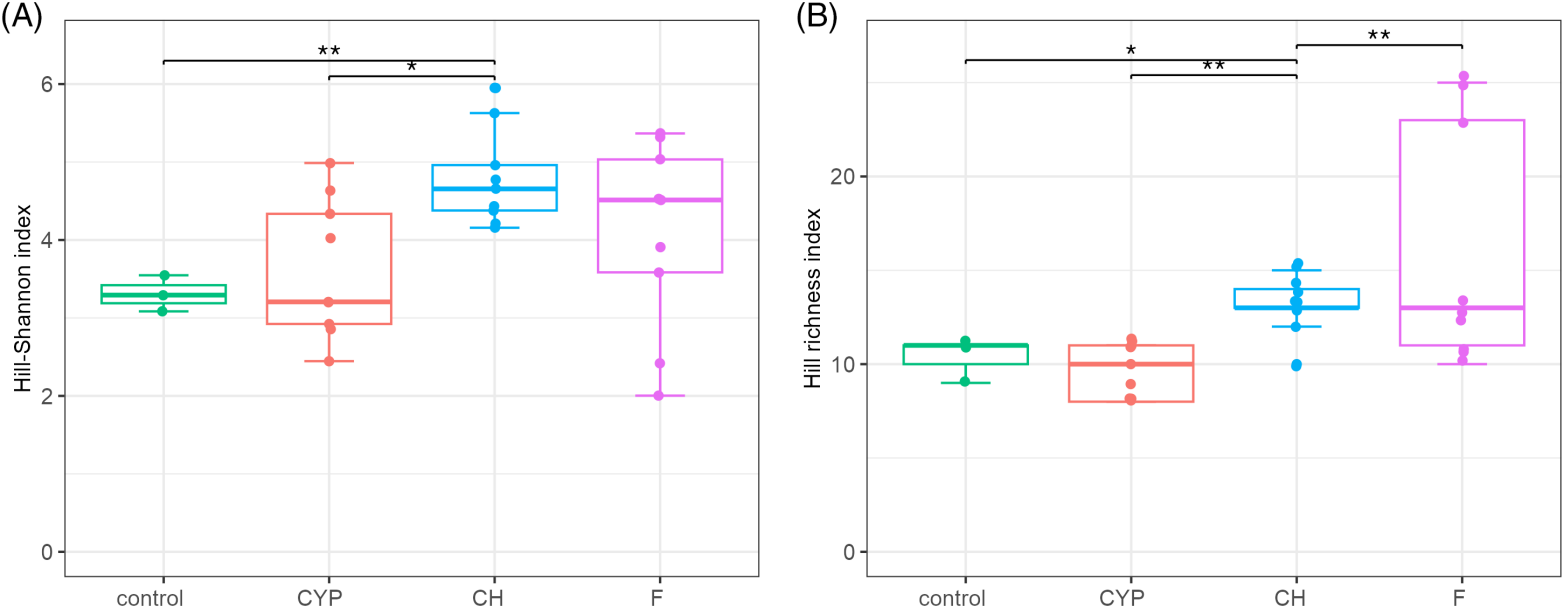
Biodiversity of cereal leaf beetle (CLB) larvae bacterial community at the genus level, depending on the variable ‘the active substance of insecticide.’ (A) alpha diversity distribution of each group according to the Hill‐Shannon index, (B) diversity distribution of each group according to the Hill‐richness index (the vertical lines indicate the range excluding the outliers, the middle lines represent the median value, the boxes represent the upper and lower quartile values). Control – no insecticide‐treated larvae, CYP, cypermethrin, CH, chlorpyrifos, F, flonicamid. Significant differences between groups are marked with an asterisk according to the following significant codes for *p*‐value: ***0.001, **0.01, *0.05, 0.1, 1.

Results of the Kruskal Wallis test for alpha diversity indicated that the variable ‘the active substance of insecticide’ generates significant differences in the biodiversity of bacterial genera at *p* < 0.05 (Table 1). According to the results of the Wilcoxon test, the biodiversity of chlorpyrifos‐treated insects was statistically significantly different from the control group of insects (*p* < 0.01) and cypermethrin‐treated larvae (*p* < 0.05) (Table S4). The obtained distribution of Hill Shannon index values showed that chlorpyrifos‐treated larvae were characterized by the highest bacterial genera diversity with respect to the number of dominant bacterial genera, as well as the structure of the microbiome. The effect of insecticide on the diversity of bacterial genera was less noticeable in the variant where cypermethrin was used (Figure 6A). The highest number of rare bacterial genera was recorded in flonicamid‐treated larvae, in turn, the lowest in the control group of insects, as shown using Hill richness index (Figure 6B).

**FIGURE 7:**
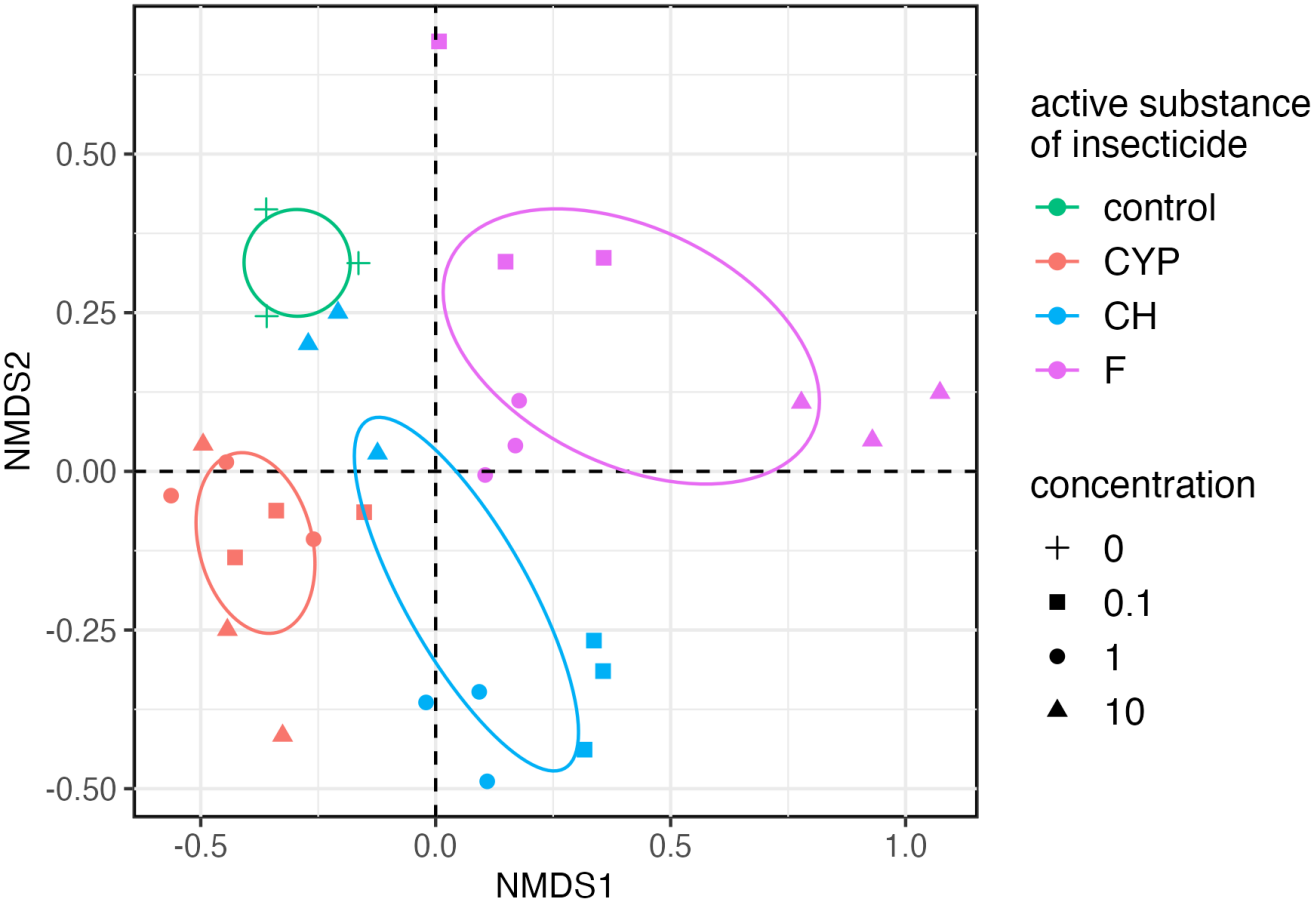
Non‐metric multidimensional scaling (NMDS) of cereal leaf beetle (CLB) larvae associated bacterial community based on Bray–Curtis dissimilarity with ellipses representing 95% confidence intervals. Data are for CLB larvae treated with three classes of insecticides containing the following active substances: cypermethrin (CYP), chlorpyrifos (CH) and flonicamid (F) at the concentrations 0.1, 1 and 10 μg/mL. Control – no insecticide‐treated larvae. The colour and shape were interpreted in the legend.

Obtained results indicated that the variable ‘the active substance of insecticide’ (*p* < 0.001) might take part in shaping up the CLB larvae‐associated bacterial microbiome on a genus level (Table 1). To compare the community structure between control and insecticide‐treated samples, the distance matrices were calculated using the Bray–Curtis dissimilarity index and were visualized using the first two components of the NMDS procedure. Analysis of data on bacteria of CLB larvae explained the relationship between the active substance of insecticide and the applied concentration. There is a clear clustering of probes for each active substance used, in contrast to the variable ‘concentration’, where no clear clustering of probes is evident (Figure 7).

**TABLE 1:** Alpha and beta diversity statistics regarding the variable ‘the active substance of insecticide’ and its ‘concentration’.

	*p*‐value	
	Variable ‘the active substance of insecticide’	Variable ‘concentration’
*α*‐diversity		
Kruskal‐Wallis test	0.013*	0.570
*β*‐diversity		
PERMANOVA	0.001***	0.037*

*Note*: Significant codes for *p*‐value: ***0.001, **0.01, *0.05, 0.1, 1.

#### Analysis of the biodiversity of CLB larvae‐associated bacteria in reference to the used concentration of the active substance of insecticide

**FIGURE 8:**
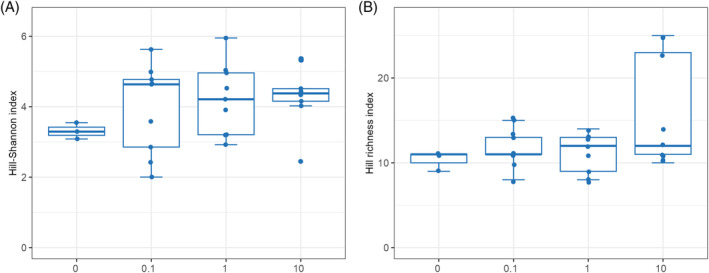
Biodiversity of cereal leaf beetle (CLB) larvae bacterial community at the genus level, depending on the variable ‘concentration of the active substance of insecticide’ (0.1, 1 and 10 μg/mL). (A) Alpha diversity distribution of each group according to the Hill‐Shannon index, (B) diversity distribution of each group according to the Hill‐richness index (the vertical lines indicate the range excluding the outliers, the middle lines represent the median value, the boxes represent the upper and lower quartile values). 0 means that no insecticide treatment was used.

Kruskal‐Wallis (Table 1) and Wilcoxon tests (Table S5) for alpha diversity indicated that the variable ‘concentration of the active substance of insecticide’ had no statistical effect on the biodiversity of bacterial genera. According to the distribution of the Hill Shannon index, an average higher bacterial biodiversity was recorded in larvae treated for each of the applied concentrations of the active substance of insecticide, compared to the control insects (no insecticide treatment) (Figure 8A).

The distance matrices calculated by using the Bray‐Curtis index showed that all concentrations of the active substance of insecticide (*p* < 0.05) might alter the structure of the CLB larvae‐associated microbiome on a genus level (Table 1). The highest number of rare bacterial genera was recorded in larvae treated with the highest concentration of the active substance of insecticide (10 μg/mL), and the lowest in the control group of insects (no insecticide treatment), as shown using the Hill richness index (Figure 8B).

### Insecticide exposure affects microbiota function in CLB larvae

#### Bacteria‐derived acetyl‐CoA C‐acetyltransferase may play an important role in the reaction to insect treatment with flonicamid and chlorpyrifos

In silico prediction approach was used to determine probable functions of the bacterial communities in CLB larvae in response to insecticide treatment through the Picrust2 software package, which implements 16S rDNA sequence data to make predictions of the metagenome function. The predicted protein‐coding genes were categorized by function using the KEGG Orthology (KO). A high abundance of predicted functions related to acetyl‐CoA C‐acetyltransferase (K00626), pyruvate dehydrogenase E2 component (K00627), tRNA dimethylallyltransferase (K00791), nitrite reductase (NADH) large subunit (K00362) and [glutamine synthetase] adenylyltransferase (K00982) was noted for all insect treatments. Higher prediction function of bacteria‐derived acetyl‐CoA C‐acetyltransferase was noted in flonicamid‐ and chlorpyrifos‐treated larvae (except for larvae treated with chlorpyrifos at a concentration of 10 μg/mL), on the other hand, lower in insects exposed to cypermethrin, than for the control insects (no insecticide treatment) (Figure S3). In turn, in cypermethrin‐treated larvae higher prediction function of tRNA dimethylallyltransferase was noted.

#### Bacteria‐derived glycine/D‐amino acid oxidase may be important in insecticide‐treated CLB larvae

To better understand the important role of the CLB larvae‐associated bacteria functional composition prediction was done by PICRUSt2 based on cluster of orthologous genes (COGs). The results showed that the most functional prediction enzyme is glycine/d‐amino acid oxidase (deaminating) (COG0665 related to category E‐amino acid [transport and metabolism]) and DNA‐binding transcriptional regulator, IcIR family (COG1414). The proportion of occurrence of glycine/D‐amino acid oxidase (deaminating) was higher in CLB larvae exposed to tested three classes of insecticides (except for larvae treated with flonicamid at a concentration of 0.1 μg/mL), compared to the no insecticide treated‐insects (Figure S4).

## DISCUSSION

Bacteria might be excellent targets for reducing the harm of herbivorous insects, as they influence a number of life processes of their insect host, including nutrition, immune system, and xenobiotic detoxification (Giglio et al., 2021; Wielkopolan & Obrępalska‐Stęplowska, 2016). The alternations in the bacterial community can affect insect life processes dependent on the microbiota. To date, no studies have been done to explain how insecticides affect the bacterial community of CLB larvae, whether associated bacteria can reduce the efficiency of insecticides, and how the presence of bacteria affects the survival of insecticide‐treated larvae.

In this study, it was shown that bacterial microbiome increases the chances of survival of CLB larvae after insecticide application. The reduction of bacteria by antibiotics significantly reduced the survival of the larvae exposed to cypermethrin and chlorpyrifos, especially at the highest concentration of active substance (10 μg/mL), compared to insects treated with insecticide alone. The highest insect mortality was recorded after the application of cypermethrin, perhaps, because of its antibacterial properties, which may have had a negative effect on certain genera of bacteria (Gangola et al., 2018). In turn, the depletion of the insect‐associated bacteria by AB followed by the flonicamid treatment slightly increased insect mortality. It can be hypothesized that bacteria take a small part in reducing the harmful effect of flonicamid or the effect of flonicamid is shifted in time. Research by Zhang et al. (2023) confirmed that the gut microbiota can play a crucial role in pesticide resistance because AB‐treated silkworm (*Bombyx mori*) was more vulnerable to chlorpyrifos. Similarly, (Pietri et al., 2018; Pietri & Liang, 2018) reported that AB treatment resulted in higher mortality of the indoxacarb‐treated *Blatella germanica*. In turn, Soh and Veera Singham (2022) noted that rifampicin‐treated *Cimex hemipterus* was more susceptible to fenitrothion and imidacloprid.

Previous results indicated that the developmental stage, host plant and locations from which insects are collected affected the CLB microbiome and that *Wolbachia* and *Rickettsia* genera, constituting the core microbiome, may be involved in life processes vital for the CLB (Wielkopolan et al., 2021). In the current study, it was shown that different genera of bacteria may be involved in the detoxification process of different insecticides with different mechanisms of action. After insecticide application, the relative abundance of *Pantoea* sharply decreased, while *Stenotrophomonas* and *Acinetobacter* significantly increased, compared to a control group of insects. In cypermethrin‐treated larvae also a significant decrease in the abundance of bacteria of the genus *Pseudomonas* was noted. In addition, changes in the dominance of bacterial genera were recorded. In cypermethrin‐treated larvae, the dominant genus of bacteria was *Pantoea* and *Lactococcus*, while in flonicamid‐ and chlorpyrifos‐treated larvae it was *Pseudomonas*. It is likely that the genera of bacteria for which an increase in abundance was recorded are tolerant to the insecticides used, or maybe they are able to degrade insecticides, making them less effective. The above‐mentioned genera of bacteria are known to degrade insecticides (Gilani et al., 2016; Ibrahim et al., 2021; Jaffar et al., 2022; Özdal & Algur, 2020, 2022). For instance, Akami et al. (2019) demonstrated that genera including *Acinetobacter*, *Citrobacter*, *Pseudomonas*, *Burkholderiales* (unclassified genera) are potentially involved in the increased tolerance of their insect host—*Callosobruchus maculatus* (Coleoptera, Chrysomelidae) against the dichlorvos insecticide (DDVP).

Several insect enzymes (including hydrolase, esterase, acetylcholinesterase, carboxylesterase, laccase, glutathione S‐transferase and cytochrome P450) are linked to the degradation of insecticides (Jaffar et al., 2022). Also, enzymes of insect‐associated microbes might contribute to the degradation of various pesticides and metabolize them into less toxic substances. Microbiota uses insecticides as a source of carbon, sulfur and energy (Jaffar et al., 2022; Mohammadi et al., 2021). Insect‐associated microbes can also indirectly influence insect host resistance to insecticide through the activation of the detoxification of the enzymes or immune system in the host (Liu & Guo, 2019; Zhao et al., 2022). For instance, *Stenotrophomonas maltophilia* enhanced *Bombyx mori* insecticide resistance not by directly degrading chlorpyrifos, but indirectly by promoting the activity levels of acetylcholinesterase in hosts (Chen et al., 2020; Zhao et al., 2022). In turn, Tang et al. (2020) reported that the following genera: *Wolbachia*, *Arsenophonus*, *Acinetobacter*, *Staphylococcus* may affect insecticide degradation by regulating the expression of the insect host's genes encoding glutathione S‐transferase and cytochrome P450. Here, we indicated the putative microbial enzymes associated with xenobiotic degradation in CLB larvae. In our research, a higher prediction function of the acetyl‐CoA C‐acetyltransferase was noted in flonicamid‐ and chlorpyrifos‐treated larvae (except for larvae treated with chlorpyrifos at a concentration of 10 μg/mL) than for the control group of insects (no insecticide treatment). It was documented previously that acetyl‐CoA C‐acetyltransferase takes part in bacterial xenobiotic degradation (Dada et al., 2018). In turn, in larvae exposed to cypermethrin, a higher prediction function of tRNA dimethylallyltransferase was noted, compared to other insect treatments. The proportion of occurrence of glycine/D‐amino acid oxidase (deaminating) was higher in CLB larvae exposed to all three tested classes of insecticides. It suggests the potential involvement of CLB associated microbiota in xenobiotic degradation. Insecticide exposure can also indirectly affect the structure and function of insect‐associated microbes, through modification of functional and morphological conditions of the insect gut. Such modifications could bring out a niche competition among different bacterial genera for nutrient sources, changing their colonization ability (Giglio et al., 2021). Modification observed in the microbiota structure of insects may also result from antagonistic interactions among bacteria with different metabolic requirements.

The symbiont‐mediated insecticide susceptibility in CLB larvae may not be caused by the presence of single taxa or species but rather depends on the diversity and complex interactions of the microbiota and their metabolic dynamics that can be disrupted or altered by insecticide treatment. In the present study, based on the alpha diversity analysis, distinctive differences in the richness and diversity of bacterial communities were observed among insecticide‐treated larvae and control populations. It is unclear why insecticide‐treated CLB larvae are characterized by higher biodiversity and richness of bacterial genera compared to a control group of insects. Similar results were noted by Juma et al. (2020). It can be therefore concluded that (i) applied insecticides may have led to a reduction of the abundance of insecticide‐susceptible bacterial taxa, in favour of the insecticide‐tolerant bacterial taxa, allowing them to proliferate, (ii) some bacteria are able to degrade insecticide and utilize them as a carbon source and may also have benefited from the reduced growth of insecticide‐sensitive bacterial genera (Aislabie & Lloyd‐Jones, 1995; Muturi et al., 2021) (iii) the use of insecticides may have led to a reduction in the dominant bacterial taxa in favour of the rare ones. The different level of biodiversity of bacterial genera in insecticide‐treated larvae, compared to control may suggest the insect's adaptive plasticity to the insecticide used.

## CONCLUSIONS

In conclusion, the effectiveness of insecticides may depend largely on the bacterial symbionts associated with CLB larvae. According to our research, bacterial microbiome increases the chances of survival of CLB larvae after cypermethrin and chlorpyrifos application. In turn, the reduction of the microbiome slightly increased insect mortality after flonicamid treatment. It can be hypothesized that the bacterial microbiome contributed little to flonicamid resistance, or the effect of flonicamid was time‐shifted. Genera of bacteria whose abundance significantly increased after insecticide application can be tolerant to the insecticide used or perhaps their direct (metabolism of the insecticides) or indirect effect reduces the effectiveness of the insecticides used. *Acinetobacter*, *Stenotrophomona*s, *Lactococcus* and *Pseudomonas* are the genera of bacteria potentially involved in limiting the harmful effect of tested insecticides in CLB larvae (depending on the class of insecticide). The class of insecticide (and its mode of action) had an impact on the structure, biodiversity and richness of bacterial genera as well as the aforementioned larvae survival rate. The effect of insecticide on the diversity of bacterial genera was less noticeable in the variant using cypermethrin. The highest number of rare bacterial genera was recorded in the flonicamid‐treated larvae. Probably the use of this insecticide may have led to a reduction in the dominant bacterial taxa in favour of the rare ones. The present study reports the first indication of the putative role of microbial enzymes in the reduction in the effectiveness of insecticides against CLB larvae. In the era of the withdrawal of many insecticides from use, including those against CLB larvae, it is important to develop new alternative methods of pest control. The results presented in this article may constitute the basis for developing new possibilities to reduce the damage caused by CLB larvae and for isolating efficient microbial species to protect the agro‐ecosystem (bioremediation).

## AUTHOR CONTRIBUTIONS

**Beata Wielkopolan:** Conceptualization (lead); data curation (equal); formal analysis (equal); funding acquisition (lead); investigation (equal); methodology (lead); visualization (equal); writing – original draft (lead); writing – review and editing (lead). **Alicja Szabelska‐Beręsewicz:** Conceptualization (supporting); data curation (equal); methodology (supporting); visualization (lead); writing – original draft (equal); writing – review and editing (equal). **Jan Gawor:** Data curation (equal); methodology (supporting); writing – original draft (supporting); writing – review and editing (equal). **Aleksandra Obrępalska‐Stęplowska:** Conceptualization (lead); formal analysis (equal); investigation (equal); methodology (equal); resources (equal); supervision (lead); visualization (equal); writing – original draft (equal); writing – review and editing (equal).

## CONFLICT OF INTEREST STATEMENT

The authors declare no conflicts of interest.

## Supporting information


**Data S1.** Supporting Information.

## Data Availability

Data generated during this study was deposited in NCBI SRA database under BioProject ID: PRJNA1036017 (https://submit.ncbi.nlm.nih.gov/about/sra).
