# Supplementary material for: Cereal leaf beetle‐associated bacteria enhance the survival of their host upon insecticide treatments and respond differently to insecticides with different modes of action
Source: Environ Microbiol Rep. 2024 Apr 21;16(2):e13247. doi: 10.1111/1758-2229.13247 (PMC11033208; doi:10.1111/1758-2229.13247)
Supplement: Supplementary file 1 — Data S1. Supporting Information. [file EMI4-16-e13247-s001.docx]

Supplementary materials

**Cereal leaf beetle-associated bacteria enhance the survival of their host upon insecticide treatments and respond differently to insecticides with different modes of action**

Beata Wielkopolan^1^, Alicja Szabelska-Beręsewicz^2^, Jan Gawor^3^, Aleksandra Obrępalska-Stęplowska^4^

^1^ Department of Monitoring and Signaling of Agrophages, Institute of Plant Protection–National Research Institute, Poznan, Poland

^2^ Department of Mathematical and Statistical Methods, Poznań University of Life Sciences, Poland

^3^ DNA Sequencing and Synthesis Facility, Institute of Biochemistry and Biophysics, Polish Academy of Sciences, Poland

^4^ Department of Molecular Biology and Biotechnology, Institute of Plant Protection–National Research Institute, Poznan, Poland

Correspondence to: Aleksandra Obrępalska-Stęplowska, Department of Molecular Biology and Biotechnology, Institute of Plant Protection–National Research Institute, 20 Węgorka St, 60‑318 Poznan, Poland. Email: [olaob@o2.pl](mailto:olaob@o2.pl)

**TABLE S1.** Data on the number of reads obtained by Next Generation Sequencing (NGS) analysis of 16S rRNA gene at the V3-V4 hypervariable region of bacteria associated with CLB larvae treated with insecticides containing following active substances: chlorpyrifos (CH), cypermethrin (CYP), flonicamid (F) at three concentrations (0.1, 1 and 10 µg/mL). Control - insecticide-untreated larvae, Blank - a control for monitoring sample contamination of environmental bacterial DNA.

| Sample names | Treatment | Replicate | Number of raw reads | Filtered forward reads | Filtered reverse reads | Merged reads | Reads without chimeras |
| --- | --- | --- | --- | --- | --- | --- | --- |
| S4922Nr1 | Control | 1 | 67246 | 30827 | 30827 | 30663 | 30405 |
| S4922Nr2 |  | 2 | 71378 | 32911 | 32911 | 32700 | 32443 |
| S4922Nr3 |  | 3 | 37302 | 17183 | 17183 | 17019 | 16917 |
| S4922Nr4 | CYP_0.1µg/mL | 1 | 79026 | 36228 | 36228 | 35942 | 35542 |
| S4922Nr5 |  | 2 | 47602 | 21800 | 21800 | 21660 | 21385 |
| S4922Nr6 |  | 3 | 46432 | 21201 | 21201 | 21027 | 20779 |
| S4922Nr7 | CYP_1µg/mL | 1 | 66516 | 30345 | 30345 | 30182 | 29977 |
| S4922Nr8 |  | 2 | 73268 | 32964 | 32964 | 32773 | 31991 |
| S4922Nr9 |  | 3 | 77850 | 35541 | 35541 | 35327 | 34618 |
| S4922Nr10 | CYP_10µg/mL | 1 | 76990 | 34820 | 34820 | 34601 | 34331 |
| S4922Nr11 |  | 2 | 86714 | 39486 | 39486 | 39257 | 39113 |
| S4922Nr12 |  | 3 | 83852 | 38221 | 38221 | 38058 | 37501 |
| S4922Nr13 | CH_0.1µg/mL | 1 | 75314 | 34720 | 34720 | 34010 | 33715 |
| S4922Nr14 |  | 2 | 71550 | 32883 | 32883 | 31982 | 30726 |
| S4922Nr15 |  | 3 | 81428 | 37453 | 37453 | 36754 | 36500 |
| S4922Nr16 | CH_1µg/mL | 1 | 53280 | 24764 | 24764 | 23538 | 23011 |
| S4922Nr17 |  | 2 | 85960 | 39464 | 39464 | 35946 | 35212 |
| S4922Nr18 |  | 3 | 85348 | 39318 | 39318 | 39010 | 28826 |
| S4922Nr19 | CH_10µg/mL | 1 | 92658 | 42591 | 42591 | 42336 | 41991 |
| S4922Nr20 |  | 2 | 71140 | 32361 | 32361 | 32191 | 31887 |
| S4922Nr21 |  | 3 | 94780 | 43094 | 43094 | 42421 | 42105 |
| S4922Nr22 | F_0.1µg/mL | 1 | 74362 | 34475 | 34475 | 34225 | 33793 |
| S4922Nr23 |  | 2 | 77920 | 35531 | 35531 | 35312 | 35016 |
| S4922Nr24 |  | 3 | 54494 | 25562 | 25562 | 25344 | 25111 |
| S4922Nr25 | F_1µg/mL | 1 | 53376 | 24220 | 24220 | 23994 | 23871 |
| S4922Nr26 |  | 2 | 85240 | 39305 | 39305 | 38861 | 37847 |
| S4922Nr27 |  | 3 | 59042 | 27215 | 27215 | 27019 | 26929 |
| S4922Nr28 | F_10µg/mL | 1 | 83498 | 38312 | 38312 | 38067 | 37834 |
| S4922Nr29 |  | 2 | 73586 | 33917 | 33917 | 33458 | 32851 |
| S4922Nr30 |  | 3 | 69198 | 32135 | 32135 | 31508 | 31185 |
| S4922Nr31 | Blank | 1 | 184 | 75 | 75 | 67 | 67 |


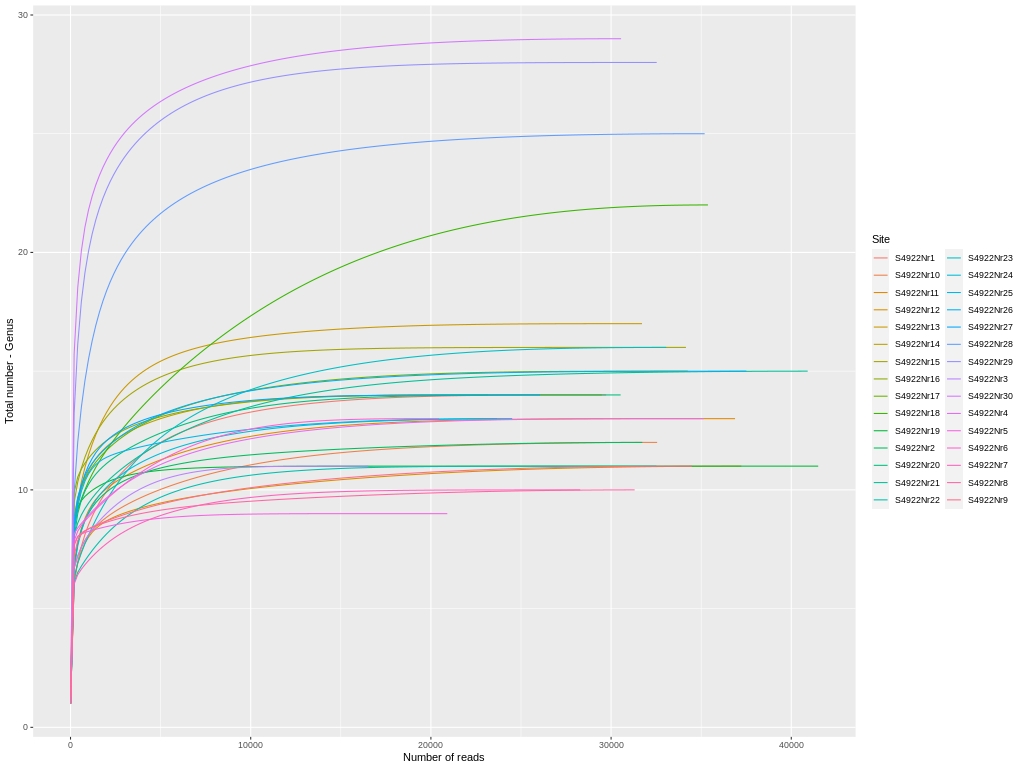


**FIGURE S1.** Rarefaction curves of all samples from the study groups. Each line represents each replicate used in respective study groups. S4922Nr 1-3 - insecticide-untreated larvae (control); 4-6 - cypermethrin 0.1 µg/mL; 7-9 - cypermethrin 1 µg/mL; 10-12 - cypermethrin 10 µg/mL; 13-15 - chlorpyrifos 0.1 µg/mL; 16-18 - chlorpyrifos 1 µg/mL; 19-21 - chlorpyrifos 10 µg/mL; 22-24 - flonicamid 0.1 µg/mL; 25-27 - flonicamid 1 µg/mL; 28-30 - flonicamid 10 µg/mL.

**TABLE S2.** Significant differences between individual pairs of levels of variables “the active substance of insecticide” (CH, chlorpyrifos; CYP, cypermethrin; F, flonicamid) and its “concentration” (0.1, 1 and 10 µg/mL) were calculated jointly using Multiple Comparisons of Means (Tukey Contrasts). Control - insecticide-untreated larvae. Significant codes for p-value: ***0.001, **0.01, *0.05,.0.1, ̣ˈ1

| **Pairs of levels**  **(the active substance of insecticide -concentration)** | **Genus** | |
| --- | --- | --- |
|  | **estimate** | **p-value** |
| CH_0.1-control | 1.13831 | 0.4494 |
| CH_1-control | 1.09810 | 0.5133 |
| CH_10-control | 0.56060 | 0.9873 |
| CYP_0.1-control | 0.03125 | 1.0000 |
| CYP_1-control | -0.58740 | 0.9866 |
| CYP_10-control | -0.31807 | 0.9999 |
| F_0.1-control | 0.48262 | 0.9957 |
| F_1-control | 0.73173 | 0.9270 |
| F_10-control | 4.16759 | <0.001*** |
| CH_1-CH_0.1 | -0.04021 | 1.0000 |
| CH_10-CH_0.1 | -0.57770 | 0.9802 |
| CYP_0.1-CH_0.1 | -1.10706 | 0.4948 |
| CYP_1-CH_0.1 | -1.72571 | 0.0376* |
| CYP_10-CH_0.1 | -1.45638 | 0.1267 |
| F_0.1-CH_0.1 | -0.65569 | 0.9535 |
| F_1-CH_0.1 | -0.40658 | 0.9985 |
| F_10-CH_0.1 | 3.02928 | <0.001*** |
| CH_10-CH_1 | -0.53749 | 0.9886 |
| CYP_0.1-CH_1 | -1.06685 | 0.5579 |
| CYP_1-CH_1 | -1.68550 | 0.0497* |
| CYP_10-CH_1 | -1.41617 | 0.1608 |
| F_0.1-CH_1 | -0.61548 | 0.9704 |
| F_1-CH_1 | -0.36637 | 0.9994 |
| F_10-CH_1 | 3.06950 | <0.001*** |
| CYP_0.1-CH_10 | -0.52936 | 0.9916 |
| CYP_1-CH_10 | -1.14801 | 0.4981 |
| CYP_10-CH_10 | -0.87867 | 0.8071 |
| F_0.1-CH_10 | -0.07798 | 1.0000 |
| F_1-CH_10 | 0.17112 | 1.0000 |
| F_10-CH_10 | 3.60699 | <0.001*** |
| CYP_1-CYP_0.1 | -0.61865 | 0.9809 |
| CYP_10-CYP_0.1 | -0.34932 | 0.9997 |
| F_0.1-CYP_0.1 | 0.45137 | 0.9974 |
| F_1-CYP_0.1 | 0.70048 | 0.9444 |
| F_10-CYP_0.1 | 4.13635 | <0.001*** |
| CYP_10-CYP_1 | 0.26934 | 1.0000 |
| F_0.1-CYP_1 | 1.07002 | 0.6008 |
| F_1-CYP_1 | 1.31913 | 0.2883 |
| F_10-CYP_1 | 4.75500 | <0.001*** |
| F_0.1-CYP_10 | 0.80069 | 0.8794 |
| F_1-CYP_10 | 1.04980 | 0.5932 |
| F_10-CYP_10 | 4.48566 | <0.001*** |
| F_1-F_0.1 | 0.24911 | 1.0000 |
| F_10-F_0.1 | 3.68497 | <0.001*** |
| F_10-F_1 | 3.43587 | <0.001*** |


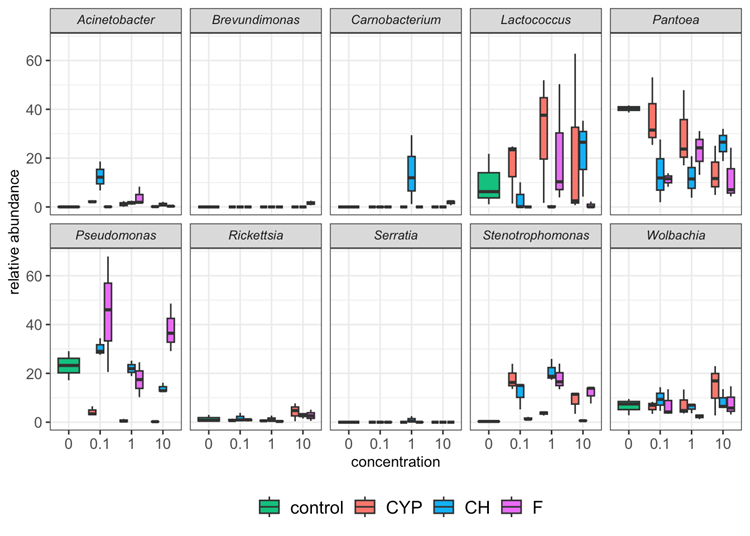


**FIGURE S2.** Boxplots visualizing the average relative abundance of bacterial genera depending on the variable “the active substance of insecticide” (CYP, cypermethrin; CH, chlorpyrifos; F, flonicamid) and its “concentration” (0.1; 1; 10 µg/mL). Only the taxa with relative abundance >1% were listed. Control - insecticide-untreated larvae.

**TABLE S3.** Results of differential abundance analysis done with DESeq2 method. Each pair of columns present log2FoldChange and adjusted p-value with the Bonferroni method (FDR) for comparison of considered active substances of insecticides (CYP, cypermethrin; CH, chlorpyrifos; F, flonicamid) and their concentration (0.1; 1; 10 µg/mL) with respect to the control group of insects (insecticide-untreated larvae).

| Bacterial genera/treatment of insects | CH 0.1 | | CH 1 | | CH 10 | | CYP 0.1 | | CYP 1 | | CYP 10 | | F 0.1 | | F 1 | | F 10 | |
| --- | --- | --- | --- | --- | --- | --- | --- | --- | --- | --- | --- | --- | --- | --- | --- | --- | --- | --- |
|  | log2FC | FDR | log2FC | FDR | log2FC | FDR | log2FC | FDR | log2FC | FDR | log2FC | FDR | log2FC | FDR | log2FC | FDR | log2FC | FDR |
| *Acinetobacter* | 6.791 | 0.000 | 4.532 | 0.000 | 4.104 | 0.002 | 5.109 | 0.000 | 5.050 | 0.000 | 2.238 | 0.193 | 1.880 | 0.373 | 5.673 | 0.000 | 2.241 | 0.135 |
| *Stenotrophomonas* | 3.749 | 0.000 | 5.143 | 0.000 | 0.241 | 1.000 | 5.203 | 0.000 | 3.818 | 0.000 | 4.913 | 0.000 | 2.868 | 0.000 | 5.004 | 0.000 | 4.846 | 0.000 |
| *Ralstonia* | -8.328 | 0.000 | -4.394 | 0.013 | 1.640 | 1.000 | -1.896 | 0.972 | -1.059 | 1.000 | 0.454 | 1.000 | -4.235 | 0.079 | -5.414 | 0.006 | -0.575 | 0.843 |
| *Pantoea* | -3.172 | 0.004 | -3.059 | 0.007 | -1.789 | 0.551 | -1.227 | 0.972 | -0.555 | 1.000 | -1.194 | 0.868 | -1.876 | 0.265 | -2.158 | 0.087 | -2.922 | 0.016 |
| *Sanguibacter* | 6.475 | 0.105 | 0.000 | 1.000 | 0.000 | 1.000 | 0.000 | 1.000 | 0.000 | 1.000 | 0.000 | 1.000 | 6.139 | 0.186 | 7.456 | 0.030 | 8.293 | 0.017 |
| *Paenibacillus* | -4.454 | 0.115 | -5.815 | 0.030 | -1.330 | 1.000 | -3.962 | 0.396 | -7.204 | 0.012 | -7.455 | 0.011 | 1.773 | 1.000 | 0.099 | 1.000 | 1.081 | 0.825 |
| *Saccharibacillus* | 7.841 | 0.115 | 6.208 | 0.279 | 0.000 | 1.000 | 0.000 | 1.000 | 0.000 | 1.000 | 2.311 | 1.000 | 2.363 | 1.000 | 0.000 | 1.000 | 5.521 | 0.312 |
| *Lactococcus* | -4.466 | 0.181 | -7.492 | 0.007 | -0.636 | 1.000 | -0.999 | 1.000 | 0.778 | 1.000 | 1.245 | 1.000 | -14.663 | 0.000 | -0.257 | 1.000 | -4.472 | 0.180 |
| *Pseudomonas* | -1.252 | 0.422 | -1.238 | 0.361 | -1.706 | 0.537 | -3.389 | 0.000 | -5.353 | 0.000 | -6.724 | 0.000 | 1.338 | 0.437 | -1.575 | 0.199 | 0.126 | 0.966 |
| *Exiguobacterium* | 4.188 | 0.438 | 4.496 | 0.361 | 0.000 | 1.000 | 0.000 | 1.000 | 0.000 | 1.000 | 0.000 | 1.000 | 3.787 | 0.780 | 4.496 | 0.423 | 9.938 | 0.009 |
| *Massilia* | 5.324 | 0.438 | 0.000 | 1.000 | 0.000 | 1.000 | 0.000 | 1.000 | 0.000 | 1.000 | 1.178 | 1.000 | 0.000 | 1.000 | 2.414 | 1.000 | 9.626 | 0.050 |
| *Chryseobacterium* | 3.683 | 0.579 | 3.878 | 0.478 | 1.664 | 1.000 | 1.881 | 1.000 | 1.592 | 1.000 | 3.717 | 0.868 | 0.000 | 1.000 | 0.000 | 1.000 | 4.578 | 0.333 |
| *Bacillus* | -6.839 | 0.596 | -6.275 | 0.724 | -6.307 | 1.000 | -5.642 | 1.000 | -5.030 | 1.000 | -5.281 | 1.000 | -4.854 | 1.000 | -6.017 | 0.885 | 3.421 | 0.825 |
| *Rickettsia* | -0.747 | 0.596 | -0.486 | 0.984 | 0.773 | 1.000 | -0.817 | 1.000 | -0.287 | 1.000 | 1.949 | 0.046 | 0.036 | 1.000 | -2.089 | 0.021 | 0.493 | 0.777 |
| *Staphylococcus* | -7.016 | 0.596 | -6.452 | 0.724 | -3.352 | 1.000 | -2.261 | 1.000 | -5.207 | 1.000 | -5.458 | 1.000 | -5.031 | 1.000 | -6.194 | 0.885 | -6.254 | 0.642 |
| *Wolbachia* | -0.753 | 0.596 | -1.098 | 0.361 | -0.317 | 1.000 | -0.748 | 1.000 | 0.508 | 1.000 | 1.036 | 0.783 | 0.358 | 1.000 | -2.205 | 0.016 | -0.355 | 0.825 |
| *Curtobacterium* | 4.288 | 0.966 | 0.000 | 1.000 | 0.000 | 1.000 | 0.000 | 1.000 | 0.000 | 1.000 | 0.000 | 1.000 | 0.000 | 1.000 | 0.000 | 1.000 | 4.125 | 0.807 |
| *Leucobacter* | 3.990 | 0.966 | 0.000 | 1.000 | 0.000 | 1.000 | 0.000 | 1.000 | 0.000 | 1.000 | 0.000 | 1.000 | 0.000 | 1.000 | 0.000 | 1.000 | 0.000 | 1.000 |
| *Achromobacter* | 0.296 | 1.000 | 0.734 | 1.000 | 1.213 | 1.000 | 1.194 | 1.000 | -1.725 | 1.000 | 2.393 | 1.000 | 0.879 | 1.000 | -2.711 | 0.885 | 6.643 | 0.024 |
| *Allorhizobium-NPR* | 0.000 | 1.000 | 0.000 | 1.000 | 0.000 | 1.000 | 0.000 | 1.000 | 0.000 | 1.000 | 0.000 | 1.000 | 0.000 | 1.000 | 6.054 | 0.885 | 5.280 | 0.720 |
| *Arthrobacter* | 0.000 | 1.000 | 0.000 | 1.000 | 0.000 | 1.000 | 0.000 | 1.000 | 0.000 | 1.000 | 0.000 | 1.000 | 0.000 | 1.000 | 0.000 | 1.000 | 7.451 | 0.585 |
| *Bacteroides* | 0.000 | 1.000 | 0.000 | 1.000 | 0.000 | 1.000 | 2.533 | 1.000 | 0.000 | 1.000 | 0.000 | 1.000 | 1.995 | 1.000 | 0.000 | 1.000 | 0.000 | 1.000 |
| *Brevundimonas* | 0.000 | 1.000 | 0.000 | 1.000 | 0.000 | 1.000 | 0.000 | 1.000 | 0.000 | 1.000 | 0.000 | 1.000 | 0.000 | 1.000 | 0.000 | 1.000 | 10.848 | 0.312 |
| *Carnobacterium* | 1.194 | 1.000 | 13.529 | 0.099 | 0.000 | 1.000 | 0.000 | 1.000 | 0.000 | 1.000 | 0.000 | 1.000 | 0.000 | 1.000 | 0.000 | 1.000 | 10.861 | 0.232 |
| *Cupriavidus* | 0.000 | 1.000 | 0.000 | 1.000 | 4.760 | 1.000 | 0.000 | 1.000 | 0.000 | 1.000 | 0.000 | 1.000 | 0.000 | 1.000 | 0.000 | 1.000 | 9.543 | 0.358 |
| *Cutibacterium* | 0.000 | 1.000 | 0.000 | 1.000 | 0.000 | 1.000 | 0.000 | 1.000 | 0.000 | 1.000 | 0.000 | 1.000 | 3.614 | 1.000 | 0.000 | 1.000 | 2.172 | 0.913 |
| *Escherichia-Shigella* | 0.000 | 1.000 | 0.000 | 1.000 | 3.031 | 1.000 | 0.000 | 1.000 | 0.000 | 1.000 | 3.530 | 1.000 | 3.614 | 1.000 | 1.500 | 1.000 | 1.415 | 0.949 |
| *Flavobacterium* | 0.000 | 1.000 | 0.000 | 1.000 | 0.000 | 1.000 | 0.000 | 1.000 | 0.000 | 1.000 | 0.000 | 1.000 | 0.000 | 1.000 | 0.000 | 1.000 | 6.860 | 0.631 |
| *Microbacterium* | 0.000 | 1.000 | 0.000 | 1.000 | 0.000 | 1.000 | 0.000 | 1.000 | 0.000 | 1.000 | 0.000 | 1.000 | 4.819 | 1.000 | 0.000 | 1.000 | 6.582 | 0.631 |
| *Rhodococcus* | 0.000 | 1.000 | 0.000 | 1.000 | 0.334 | 1.000 | 0.000 | 1.000 | 0.000 | 1.000 | 0.000 | 1.000 | 0.000 | 1.000 | 1.820 | 1.000 | 5.210 | 0.720 |
| *Serratia* | 0.000 | 1.000 | 9.368 | 0.408 | 0.000 | 1.000 | 0.000 | 1.000 | 0.000 | 1.000 | 0.000 | 1.000 | 4.715 | 1.000 | 1.886 | 1.000 | 0.000 | 1.000 |
| *Terribacillus* | 0.000 | 1.000 | 0.000 | 1.000 | 0.000 | 1.000 | 0.000 | 1.000 | 0.000 | 1.000 | 0.000 | 1.000 | 2.307 | 1.000 | 0.000 | 1.000 | 1.560 | 0.949 |

**TABLE S4.** Pairwise comparisons using the Wilcoxon rank sum exact test for the Hill-Shannon index (lower-left part) and Hill-richness index (upper right part) for the active substance of insecticide. Control - insecticide-untreated larvae; CYP, cypermethrin; CH, chlorpyrifos, F, flonicamid. Values represent p-values with significant codes for p-value: ***0.001, **0.01, *0.05, .0.1, 1

| Treatment | Control | CYP | CH | F |
| --- | --- | --- | --- | --- |
| Control |  | 0.4853 | 0.0395* | 0.0891. |
| CYP | 1.000 |  | 0.0015** | 0.0045** |
| CH | 0.009** | 0.014* |  | 0.8579 |
| F | 0.209 | 0.387 | 0.340 |  |

**TABLE S5.** Pairwise comparisons using Wilcoxon rank sum exact test for Hill-Shannon index (lower-left part) and Hill-richness index (upper right part) for concentrations of the active substance of insecticide (0.1, 1 and 10 µg/mL). 0 means that no insecticide treatment was used. Values represent p-values with significant codes for p-value: ***0.001, **0.01, *0.05, .0.1, 1

| Concentration of the active substance of insecticide [µg/mL] | 0 | 0.1 | 1 | 10 |
| --- | --- | --- | --- | --- |
| 0 |  | 0.34 | 0.51 | 0.19 |
| 0.1 | 0.482 |  | 0.69 | 0.47 |
| 1 | 0.282 | 0.730 |  | 0.29 |
| 10 | 0.064. | 0.931 | 0.796 |  |


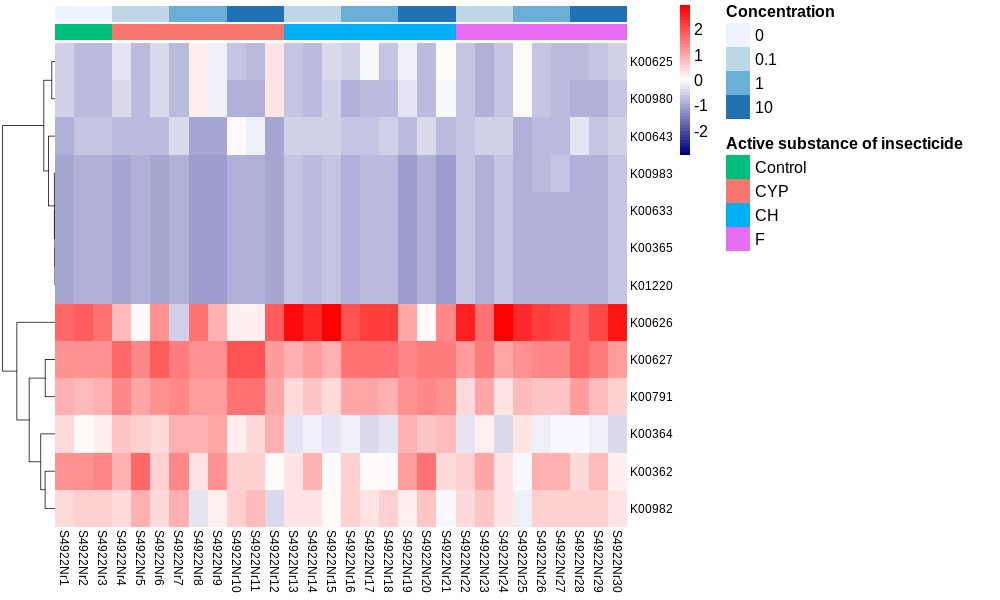


**FIGURE S3.** Heatmap of selected KEGG Orthology (KO) showing functional differences in the CLB larvae-associated microbiota, depending on the active substance of insecticide used (CYP, cypermethrin; CH, chlorpyrifos; F, flonicamid) and its concentration (0.1, 1, 10 µg/mL). Control - insecticide-untreated larvae. Bacterial genes were predicted using the PICRUSt2 algorithm followed by normalization and visualization using Microbiomeanalyst.


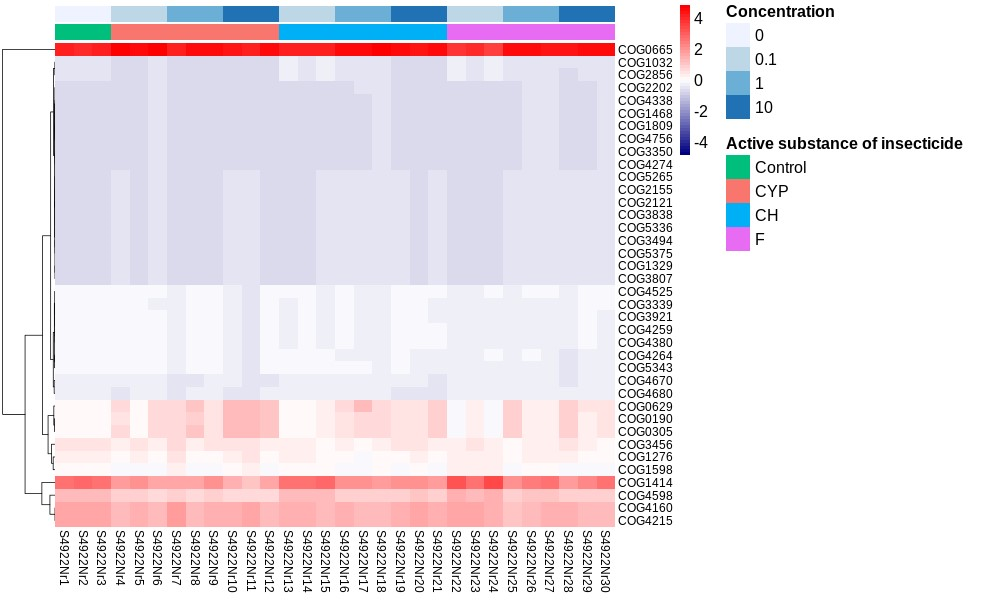


**FIGURE S4**. Heatmap of prediction function of CLB larvae-associated bacteria based on COGs (Cluster of Orthologous Genes) in response to insecticide treatment (CYP, cypermethrin; CH, chlorpyrifos; F, flonicamid) at three concentrations of the active substance of insecticide (0.1, 1, 10 µg/mL). Control - insecticide-untreated larvae. ANOVA test executed with STAMP (p<0.05 and effect size <0.45) was used to elucidate PICRUSt2 predicted COGs for visualization using Microbiomeanalyst.
